# Phylogeography of the Cactophilic *Drosophila* and Other Arthropods Associated with Cactus Necroses in the Sonoran Desert

**DOI:** 10.3390/insects2020218

**Published:** 2011-05-05

**Authors:** Edward Pfeiler, Therese A. Markow

**Affiliations:** 1 Centro de Investigatión en Alimentatión y Desarrollo, A.C., Unidad Guaymas, Apartado Postal 284, Guaymas, Sonora 85480, México; E-Mail: pfeiler@ciad.mx; 2 Division of Biological Sciences, University of California, San Diego, La Jolla, CA 92093, USA

**Keywords:** Coleoptera, Diptera, Gulf of California, historical demography, population structure, Pseudoscorpiones, speciation, vicariance

## Abstract

Studies on the population genetics, phylogenetic relationships, systematics and evolution of arthropods that inhabit necrotic tissue of cacti in the Sonoran Desert of North America are reviewed. These studies have focused upon several species of insects (orders Diptera and Coleoptera) and arachnids (order Pseudoscorpiones). For most taxa studied, little genetic structure and high dispersal ability are found in populations inhabiting the mainland and Baja California peninsula regions of the Sonoran Desert, consistent with the availability of the rotting cactus microhabitat which is patchily distributed and ephemeral. There is evidence, however, that the Gulf of California, which bisects the Sonoran Desert, has played a role in limiting gene flow and promoting speciation in several taxa, including histerid beetles, whereas other taxa, especially *Drosophila nigrospiracula* and *D. mettleri*, apparently are able to freely cross the Gulf, probably by taking advantage of the Midriff Islands in the northern Gulf as dispersal “stepping stones”. Genetic evidence has also been found for historical population expansions dating to the Pleistocene and late Pliocene in several taxa. Overall, these studies have provided important insights into how arthropods with different life history traits, but generally restricted to a necrotic cactus microhabitat, have evolved in an environmentally harsh and tectonically active region. In addition, they suggest some taxa for further, and more detailed, hypothesis driven studies of speciation.

## 1. Introduction

### 1.1. Geography of the Sonoran Desert

**Figure 1:**
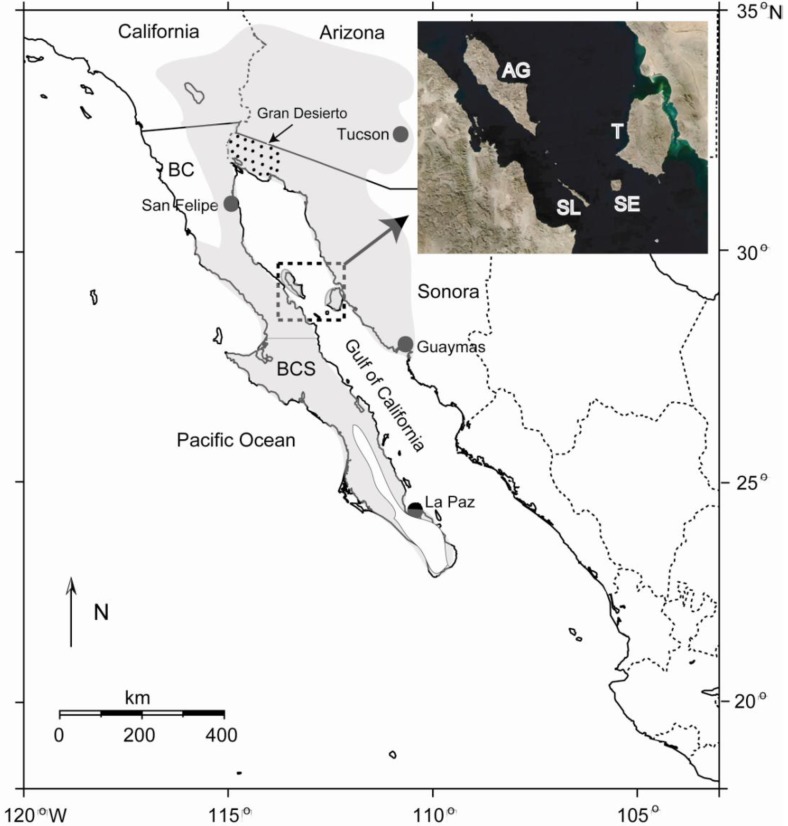
Map showing the approximate boundaries of the Sonoran Desert (shaded area) in southwestern USA and northwestern Mexico. Inset is a satellite image showing details of the four major Midriff Islands in the Gulf of California: AG, Angel de la Guarda; T, Tiburon; SE, San Esteben; SL, San Lorenzo. Stippled area at the head of the Gulf shows the region of the Gran Desierto de Altar. BC, Baja California; BCS, Baja California Sur.

The Sonoran Desert of North America encompasses approximately 260,000 km^2^ of ecologically diverse habitat in southwestern USA and northwestern Mexico [1], including the southeastern portion of California and most of southern Arizona in the USA, and the states of Baja California, Baja California Sur and Sonora in Mexico (Figure 1). Although some workers consider the peninsular portion of the Sonoran Desert a separate entity [2], herein we follow the generally accepted definition in which the peninsula is included [1]. The dynamic geological history of the region, including the still active separation of the Baja California peninsula from the mainland that began in the late Miocene [3], in addition to the vast expanse of desert vegetation, make for an ideal setting to test hypotheses on the roles of vicariance and dispersal in shaping genetic differentiation and phylogeography of desert insects and other organisms.

The Gulf of California (or Gulf; also known as the Sea of Cortez), which formed as the Baja California peninsula separated from the mainland, represents a potential dispersal barrier for insects and other terrestrial organisms. Another potential dispersal barrier is the Gran Desierto de Altar at the head of the Gulf (Figure 1), the largest active sand dune region in North America. Although the width of the Gulf varies from ∼80–200 km, certain landscape features, particularly the Midriff Islands in the northern Gulf (Angel de la Guarda, Tiburón, San Esteben, San Lorenzo, and several smaller islands; Figure 1), provide potential dispersal “stepping stones” between the mainland and the peninsula, especially for highly vagile insects. The largest water gap in this region (between the islands of San Lorenzo and San Esteben) is only about 15 km [4]. In addition, columnar cacti utilized as host plants by a variety of cactophilic insects discussed below are found on many of these islands [5], increasing their potential to facilitate dispersal and gene flow across the Gulf.

### 1.2. Insects Associated with Cactus Necroses

Necroses (rots) of several species of large columnar and other smaller cacti provide an important microhabitat for the breeding, feeding and development of at least 34 species of arthropods in the Sonoran Desert, mostly insects, including 23 families and 10 orders [6]. Cactus necroses are created by an injury to the plant that is followed by invasion of microorganisms which then decompose the plant tissue. The availability of both water and microorganisms as food source makes cactus necroses particularly attractive to arthropods in this arid region. The most important cactus hosts are saguaro (*Carnegiea gigantea*), cardón (*Pachycereus pringlei*), senita (*Lophocereus schottii*), organ pipe (pitahaya dulce; *Stenocereus thurberi*), pitahaya agria (*S. gummosus*) and cina (*S. alamosensis*) [7].

**Figure 2:**
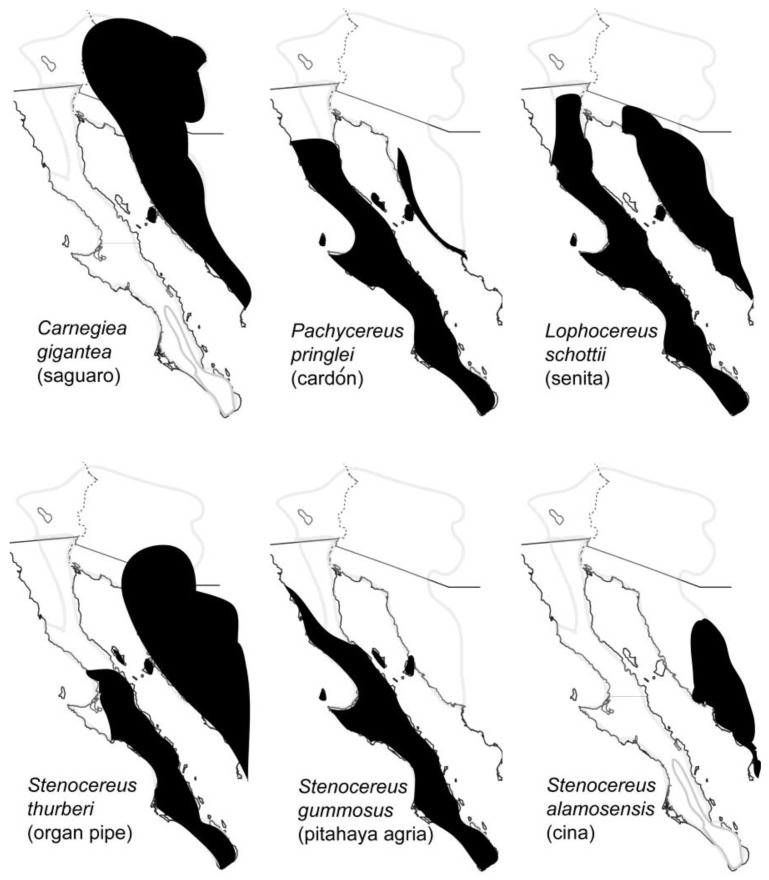
Approximate distributions of the principal cactus hosts (black shaded areas) utilized by the Sonoran Desert arthropods discussed herein based on data given in Turner *et al.* [5]. Boundary of the Sonoran Desert from Figure 1 is shown as a light gray line.

Distributions of these cactus hosts are shown in Figure 2. Two characteristics of cactus rots, their ephemeral nature and patchy distribution [7], necessitate that insects dependent on this resource be able to efficiently disperse to fresh rots that can provide the appropriate habitat for feeding and reproduction. Insect species from the orders Diptera and Coleoptera, showing a range of dispersal capabilities, are especially well represented in rotting cacti. In this review we summarize the major findings on the population genetics, systematics, dispersal and historical demography of several representatives from both insect orders, and one species of pseudoscorpion, and speculate how host plant use and landscape features of the Sonoran Desert region might have helped shape the contemporary geographic distributions of these arthropods.

## 2. Population Structure and Dispersal in Cactophilic Insects

### 2.1. The Cactophilic Diptera

The population structure and dispersal of six species of cactophilic Diptera from the Sonoran Desert have been studied. These include five species of *Drosophila* (Drosophilidae), *D. mojavensis* Patterson and Crow, *D. arizonae* Ruiz, Heed and Wasserman, *D. nigrospiracula* Patterson and Wheeler, *D. mettleri* Heed and *D. pachea* Patterson and Wheeler, and the cactus fly (Neriidae), *Odontoloxozus longicornis* (Coquillett).

#### 2.1.1. Drosophila nigrospiracula and Drosophila mettleri

Both *D. nigrospiracula* and *D. mettleri* are primarily associated with necroses of cardon cactus on the Baja California peninsula and both cardon (coastal Sonora) and saguaro on the mainland (Sonora and Arizona), although *D. mettleri* is also associated with senita necroses. The two species, however, are not in direct competition for resources as *D. nigrospiracula* develops directly in the necrotic cactus tissue whereas *D. mettleri* develops in the soil soaked with rot exudates at the base of the cactus. *Drosophila nigrospiracula* is a strong disperser [8,9] and allozyme and mitochondrial DNA (cytochrome *c* oxidase subunit I; COI) markers have shown little or no population structure for this species (as well as for *D. mettleri*) within mainland populations, and between mainland and peninsular populations [10–13]. In *D. nigrospiracula*, however, allozyme studies suggest some local differentiation in peninsular populations [11]. In *D. mettleri*, nuclear markers suggest some population structure within each geographic region, although no differentiation was found between several mainland and peninsular populations [14] in agreement with the COI data. A general lack of population structure across the Gulf seen in *D. nigrospiracula* and *D. mettleri* (Table 1), together with the fact that cardon cacti which are widely distributed on the Midriff Islands [5] support resident populations of both species [15], are observations consistent with the “stepping-stone” hypothesis of dispersal and gene flow. Migrating flies of both species, however, also have the capability to use a variety of cactus hosts [7,11,16]. Although we cannot rule out long distance dispersal, possibly wind-assisted, directly over water or via the Gran Desierto de Altar at the head of the Gulf (Figure 1), these possibilities seem unlikely given the distances involved and the harsh environmental conditions found. Columnar cacti hosts for both species are absent from the Gran Desierto de Altar (Figure 2), and potential alternate hosts for *D. mettleri*, such as prickly-pear *Opuntia* spp. [12], are also largely absent from this extremely arid region.

#### 2.1.2. Drosophila mojavensis

Genetically differentiated populations of *D. mojavensis* are found in four geographically isolated regions of southwestern USA and northwestern Mexico: (1) Mojave Desert (including the extreme northwestern Sonoran Desert) of southern California and northwestern Arizona; (2) mainland Sonora and northern Sinaloa; (3) Baja California peninsula and (4) Santa Catalina Island. Based on both morphological [17] and molecular characters [18–22] the four populations have been assigned to the subspecies *D. m. mojavensis* Patterson and Crow, *D. m. sonorensis* Castrezana, *D. m. baja* Mettler and *D. m. wrigleyi* Castrezana, respectively [17,23]. Molecular markers, including both nuclear and mitochondrial DNA, have shown that each subspecies is essentially panmictic throughout its range, but that significant structure exists between subspecies. The significant population structure found between *D. m. sonorensis* in Sonora and the peninsular *D. m. baja* (Table 1) is in contrast to the lack of structure seen for corresponding populations of *D. nigrospiracula* and *D. mettleri* separated by the Gulf, suggesting that the Midriff Islands represent a less effective dispersal route for *D. mojavensis*.

As with cardón, the principal host cacti of *D. m. baja* (pitahaya agria) and *D. m. sonorensis* (pitahaya dulce) are found on the Midriff Islands [5]. Pitahaya agria, which is present in Sonora only in a restricted region adjacent to Isla Tiburon, may itself have used these islands as “stepping stones” to disperse from the peninsula, where it is common, to mainland Sonora [4]. Because *D. m. mojavensis* and *D. nigrospiracula* show similar dispersal capability [9], with both species being essentially panmictic throughout Sonora, other explanations for genetic differentiation between peninsular and mainland flies are likely but not easily identified [21]. The observation that both mean uncorrected genetic distance (*p*-distance) [17] and *F*_st_ values [21] calculated from mitochondrial COI sequence data are lower between the peninsular and mainland populations, compared with pairwise comparisons from other two regions, suggests less genetic diversification and some gene flow across the Gulf, although nuclear DNA data suggest no gene flow between the two populations [20].

#### 2.1.3. Drosophila pachea

Populations of *D. pachea* are distributed throughout the mainland and peninsular regions of the Sonoran Desert, largely mirroring the distribution of its principal host cactus *Lophocereus schottii* (Figure 2) [24,25]. *Drosophila pachea* also utilizes the closely-related cactus *L. gatesii*, found only in a small coastal area of Baja California Sur west of La Paz [26]. *Drosophila pachea* has a lower dispersal ability compared to *D. nigrospiracula, D. mettleri* and *D. mojavensis* [9], suggesting a greater potential for local differentiation and population structure. A general lack of population structure, however, was found among most mainland populations of *D. pachea*, as well as among most peninsular populations, based on allozyme, mitochondrial DNA and microsatellite markers [10,12,25,27], in agreement with results obtained for *D. nigrospiracula*, *D. mettleri* and *D. mojavensis*. Although dispersal capability is lower in *D. pachea*, the panmixia observed within each major geographic region may be related to resource availability in that senita rots are generally found closer together compared with those of the other columnar cacti [7]. Evidence for significant structure, however, was found between mainland and peninsular populations of *D. pachea* using mitochondrial DNA [12] (Table 1). In pairwise comparisons among all mainland and peninsular populations, microsatellite data showed evidence for both population structure and panmixia, but a comparison between grouped populations from each of the two geographic areas showed low but significant structure in agreement with the mitochondrial DNA data [25]. Thus in both *D. pachea* and *D. mojavensis*, a common pattern of reduced gene flow between the mainland and peninsula compared with *D. nigrospiracula* and *D. mettleri* is found, although host plants for all four species of *Drosophila* are present on the Midriff Islands.

#### 2.1.4. Drosophila arizonae

Compared with the other species of cactophilic *Drosophila*, *D. arizonae* shows less host plant specialization, utilizing a variety of cacti for feeding and development, principally cina (*Stenocereus alamosensis*) in Sonora and *Opuntia* spp. in Baja California Sur; decaying fruits and vegetables are also utilized in urban areas [28]. Interestingly, different molecular markers have revealed contrasting patterns of population structure in *D. arizonae* in the Sonoran Desert. Analyses based on COI sequences revealed essentially no structure among two nearby populations of *D. arizonae* from the Cape Region of Baja California Sur, and among two mainland populations from southern Sonora; a lack of structure was also found between the mainland and peninsular populations [21]. Significant structure, however, was found among populations of *D. arizonae* from Tucson, Arizona and from Riverside, California (just west of the northwestern limit of the Sonoran Desert), and these two northern populations showed significant structure compared to the populations from southern Sonora and Baja California Sur [21]. In contrast to the lack of structure among mainland and peninsular populations of *D. arizonae* found with the mitochondrial COI data set, analysis of nuclear sequences showed significant structure among populations from these two regions [20]. The nuclear data set, however, confirmed the structure noted among the other populations of *D. arizonae* sampled in the COI study.

#### 2.1.5. Odontoloxozus longicornis and Dinocheirus arizonensis

The neriid cactus fly *Odontoloxozus longicornis* is considered here together with the chernetid pseudoscorpion (Order Pseudoscorpiones) *Dinocheirus arizonensis* (Banks) with which it shares an unusual symbiotic relationship. Both species are commonly found in necrotic tissue of a wide variety of cacti throughout the Sonoran Desert, with the pseudoscorpion preying upon various cactophilic insects. Although *D. arizonensis* has quite limited vagility which would tend to compromise its ability to colonize new rots, its dispersal is facilitated by attaching itself to the legs of *O. longicornis*, or other large cactophilic insects such as coleopterans in the tribe Hololeptini (see below), hitchhiking (phoretic dispersal) when the host transporter disperses to new cactus rots [29]. As with the cactophilic *Drosophila* (with the possible exception of *D. arizonae*), COI sequence analysis suggests that mainland populations of *O. longicornis* are panmictic over a wide geographic area [30]. Although only a single peninsular individual of *O. longicornis* has been sequenced [30], the haplotype of this individual was the same as the most common COI haplotype on the mainland (haplotype GY2; GenBank FJ532246), suggesting high dispersal capability between the peninsula and mainland.

Consistent with an efficient phoretic dispersal behavior, *Dinocheirus arizonensis* shows little population structure throughout the mainland Sonoran Desert [30]. Significant structure, however, was noted between peninsular populations and populations from the mainland (Table 1). Phylogenetic analysis showed that the populations of *D. arizonensis* from each of the two geographic regions resolved as distinct clades separated by a mean Kimura [31] 2-parameter (K2P) genetic distance of 2.6% [30]. Within the peninsular clade, high gene flow also was found among geographically distant populations. If phoresy was not an important dispersal mechanism for *D. arizonensis*, we would expect to find higher levels of population structure within both peninsular and mainland populations. The relatively high level of differentiation noted between peninsular and mainland populations suggests that the two lineages evolved in allopatry and possibly should be considered distinct taxa at the subspecies or species level. The sympatric occurrence of both lineages in southeastern Arizona [30], which probably occurred by secondary contact after differentiation, is consistent with this interpretation. In this scenario, phoretic dispersal, although promoting relatively high levels of gene flow on the peninsula and mainland, would be much less efficient across the Gulf, even considering the potential of the Midriff Islands to act as “stepping stones”. The effect of an attached pseudoscorpion on flight ability of *O. longicornis* has not been studied, but in all likelihood it would have a negative effect, possibly leading to the pattern of differentiation observed in *D. arizonensis*.

### 2.2. The Cactophilic Coleoptera

A large number of species belonging to the family Histeridae, hister beetles or clown beetles, are common inhabitants of rotting cacti in the Sonoran Desert. The population genetics and systematics of several of these species from three genera, *Hololepta* and *Iliotona* (subfamily Histerinae, tribe Hololeptini) and *Carcinops* (subfamily Dendrophilinae, tribe Paromalini) from widely-separated mainland and peninsular localities have been studied using both mitochondrial DNA and morphological analyses [32,33] revealing patterns of population structure, dispersal and speciation that can be directly compared with the highly vagile cactophilic dipterans which share the same necrotic cactus microhabitat. Here we assume that flight, possibly wind assisted, is the principal dispersal mechanism in this group.

#### 2.2.1. Hololepta and Iliotona

Mitochondrial DNA sequence analyses (16S rRNA and COI), combined with comparisons of morphological characters, have been conducted on seven species, or putative species, of Hololeptini associated with cactus necroses in the Sonoran Desert, three in the genus *Iliotona* [*I. beyeri* (Schaeffer), *I. dorcoides* (Lewis) and *I. cacti* (LeConte)] and four in the genus of *Hololepta* (*H. populnea* LeConte, *H. vicina* LeConte, and *Hololepta* sp. 1 and sp. 2) [33]. Based on analyses of these combined characters, *I. beyeri* was shown to be a valid species distinct from *I. dorcoides* with which it had been placed previously as a synonym [34]. In addition, phylogenetic analyses revealed sister group relationships among two pairs of species separated by the Gulf of California, *Hololepta* sp. 1 (peninsular) and *Hololepta* sp. 2 (mainland) and *I. beyeri* (peninsular) and *I. dorcoides* (mainland). Each of these species appears to be restricted to its respective geographic region, with an estimated age of divergence among each sister species pair, based on molecular clock calculations (Table 1), consistent with geological estimates for the formation of the Gulf during the late Miocene and early Pliocene [2,3,35,36]. The results suggest very limited, or a lack of dispersal of these species across the Gulf, consistent with the view that divergence and speciation in these taxa resulted from vicariance. Within the peninsular region, however, our data suggest that *I. beyeri* is panmictic [37], implying that it is a good disperser in its terrestrial habitat. Our sample of *I. beyeri* from the peninsula was collected on senita, except for a single individual found on pitahaya agria. Both cacti are present on the Midriff Islands (Figure 2).

#### 2.2.2. Carcinops

At least ten species of very small beetles (∼2–3 mm in length) in the genus *Carcinops* are known to be associated with cactus necroses in the Sonoran Desert. Of these, four have been formally described: *C. consors* (LeConte), *C. gilensis* (LeConte), *C. corticalis* (LeConte) and *C. opuntiae* (LeConte); six additional species identified using both morphological and molecular characters [32] await formal description. In our sampling of cactus necroses throughout the peninsular and mainland Sonoran Desert a large number of *Carcinops* were found. We present a brief summary of our preliminary findings on the population genetics of this group, based on analysis of COI sequences [37], which complement the findings on the other species of cactophilic arthropods described above.

Utilizing both morphological and molecular (COI) characters given in Swanson [32] we determined that most of the peninsular *Carcinops* in our sample belonged to the taxon *C. gilensis. Carcinops gilensis* is a widely distributed histerid beetle, known from southern Arizona and southern California in the USA, and from Sonora and the Baja California peninsula in Mexico. *Carcinops gilensis* is typically associated with the necrotic stems of saguaro and cardon, but California specimens were taken from necrotic barrel cactus (*Ferocactus* sp.) [32] and peninsular specimens were collected together with the Hololeptini [33], mainly from senita. Our results reveal little population structure in *C. gilensis* throughout the peninsular region and, in addition, show no significant structure between samples from the Guaymas area on the mainland and populations from the mid-peninsular region. These results suggest that *C. gilensis* is able to disperse across the Gulf, probably via the Midriff Islands. Thus, *C. gilensis* and *I. beyeri* which share the same host on the Baja California peninsula appear to show similar dispersal capabilities throughout the peninsula, but *I. beyeri* which is much larger (>10 mm in length) apparently is restricted to the peninsula.

Phylogenetic analyses also revealed a sister group relationship among two undescribed species of *Carcinops* which are geographically separated, *Carcinops* sp. 1 (peninsular) and *Carcinops* sp. 2 (mainland; also identified by Swanson [32] but not yet formally described). Mean K2P genetic distance among the two populations was 3.9%, slightly lower than the mean value of 6.0% estimated for *Hololepta* sp. 1 and sp. 2 (Table 1). As with *Hololepta* sp. 1 and sp. 2, molecular clock calculations suggest that *Carcinops* sp. 1 and sp. 2 diverged during the Pliocene (Table 1). In contrast to the results seen with *C. gilensis*, our preliminary analyses suggest a lack of gene flow across the Gulf for *Carcinops* sp. 1 and sp. 2. For comparison, Table 1 also shows that genetic distance and estimated divergence time between peninsular and mainland populations (subspecies) of the hematophagous kissing bug *Triatoma rubida* (Uhler) (Hemiptera: Reduviidae) are similar to those obtained for *Hololepta* sp.1 and sp. 2 and *Carcinops* sp.1 and sp. 2 [38].

## 3. Demographic History of Cactophilic Arthropods

One interesting question, given their sharing of the same hosts across the same areas, is whether the demographic histories of these different taxa are similar. The demographic histories of several species of cactophilic arthropods have been estimated from statistical analyses of mitochondrial DNA sequence data using the mismatch distribution [39], Bayesian skyline analysis [40], and estimation of changes in population size carried out in FLUCTUATE [41]. The results of these tests suggest that mainland populations of the neriid cactus fly *O. longicornis*, and both mainland and peninsular populations of *Dinocheirus arizonensis* which are transported by the fly during phoretic dispersal, have undergone similar historical population expansions dating to the Pleistocene [30]. Bayesian skyline analysis revealed that mainland and peninsular populations of *Drosophila pachea*, except for an isolated peninsular population in northern Baja California, also showed evidence for historical population expansions dating to the Pleistocene and late Pliocene [25]. Our data also show similar population expansions for mainland and peninsular populations of *D. nigrospiracula* [37]. Thus, two different species of cactophilic *Drosophila* which utilize different host cacti both show signatures of past population expansions. In addition, our results on peninsular *C. gilensis* show a population expansion similar to that seen in the other cactophilic arthropods mentioned above, whereas the peninsular *I. beyeri*, collected from the same senita host cactus as *C. gilensis*, showed no evidence of an expansion [37]. Additional studies on potential biological and environmental factors that may have been responsible for shaping the different demographic histories of these cactophilic arthropods promise to contribute substantially to our understanding of the ecological relationships among these arthropods and the necrotic cactus microhabitat in the Sonoran Desert.

## 4. Conclusions

Tectonic events resulting in the separation of the Pacific Plate from North American Plate, and giving rise to the Gulf of California, have had a wide-ranging impact on the evolution and speciation of animal taxa inhabiting the Sonoran Desert. We have presented a brief summary of studies on the population genetics, phylogenetic relationships and demographic histories of several species of arthropods with different life histories and dispersal abilities that inhabit a common microhabitat (cactus necroses) in the Sonoran desert. Population genetic analyses on representatives from the orders Diptera, Coleoptera and Pseudoscorpiones have generally revealed little or no population structure within each of the two main geographic regions (peninsular and mainland) for most taxa studied, consistent with ecological predictions that arthropods dependent on cactus necroses should show high dispersal ability.

The Gulf of California would appear *a priori* to be a significant dispersal barrier for Sonoran Desert insects with low dispersal ability. The Midriff Islands, however, may play an important role as a dispersal route between the peninsula and mainland by acting as “stepping stones” and promoting genetic connectivity between the two regions. Thus, organisms with low vagility might be expected at some point in time to successfully cross the Gulf, possibly assisted by wind or by rafting [42] over the relatively short distances that separate these islands. Highly vagile organisms, such as *D. nigrospiracula* and *D. mettleri*, would be even more likely to make successful crossings using the “stepping stones”. The lack of significant structure between peninsular and mainland populations of both *D. nigrospiracula* and *D. mettleri* is consistent with this scenario. Our results, however, have also shown that the Gulf apparently acts as an important dispersal barrier promoting population fragmentation and speciation in other taxa. This appears to be true for the coleopteran sister taxa *Iliotona beyeri/I. dorcoides, Hololepta* sp. 1/sp. 2 and *Carcinops* sp. 1/sp. 2, but at the same time other coleopterans with apparently low vagility, such as *C. gilensis*, appear to be able to traverse the Gulf. Vicariance also appears to have played a role in population differentiation in *Drosophila mojavensis* and the pseudoscorpion *Dinocheirus arizonensis*, as well as in the hematophagous *Triatoma rubida*. Because of the abundant ecological and life history information available for most of the cactophilic species presented here, we have been able to assess biogeography and speciation as they relate to these traits. Dispersal ability and vagility, in particular, are of fundamental importance in shaping phylogeographic patterns of species, but these traits are often unknown or ignored in studies of this type [43,44].

Because only a fraction of the arthropod fauna dependent upon the necrotic cactus microhabitat has been studied to date, an important focus for future research will be to determine, using both mitochondrial and nuclear markers, if high dispersal capability within peninsular and mainland regions is a common characteristic of the fauna, and to assess the possible role of the Gulf of California and Midriff Islands in shaping genetic differentiation and evolution in these other taxa. More extensive spatial sampling for many of the taxa is essential to rigorously address the role of geological history in the patterns observed. Additionally, identification of candidate nuclear genes possibly associated with host plant adaptation, as suggested for both *Drosophila mettleri* [14] and *D. mojavensis* [22], would provide a better understanding of the molecular mechanisms underlying the abilities of these taxa to utilize this unique resource.

## Figures and Tables

**Table 1:** Summary of Kimura 2-parameter mean genetic distances (*d*), estimated divergence times in millions of years (Ma), and population structure between peninsular and mainland populations of Sonoran Desert arthropods based on COI sequence data. Interspecific comparisons are for sister species identified in phylogenetic analyses.

**Baja California Peninsula**	**Mainland**	**Mean (*d*)**	Divergence Time* (Ma)	**Pop. Structure**	**Reference**
**Diptera** (*Drosophila*)					
*D. nigrospiracula*	*D. nigrospiracula*	0.0%	-----	No	[12]
*D. mettleri*	*D. mettleri*	0.0%	-----	No	[12]
*D. mojavensis baja*	*D. m. sonorensis*	0.8%	0.4	Yes	[17]
*D. pachea*	*D. pachea*	0.9%	0.5	Yes**	[12]
*D. arizonae*	*D. arizonae*	ND	ND	No***	[21]
**Coleoptera**					
*Iliotona beyeri*	*Iliotona dorcoides*	14.6%	7.3		[33]
*Hololepta* sp. 1	*Hololepta* sp. 2	6.0%	3.0		[33]
*Carcinops* sp. 1	*Carcinops* sp. 2	3.9%	2.0		[37]
*Carcinops gilensis*	*C. gilensis*	0.1%	-----	No	[37]
**Hemiptera** (*Triatoma*)					
*T. rubida cochimiensis*	*T. r. sonoriana*	5.0%	2.5	Yes	[38]
**Pseudoscorpiones**					
*Dinocheirus arizonensis*	*D. arizonensis*	2.6%	1.3	Yes	[30]

ND: not determined;

* estimates based on 2% pairwise COI sequence divergence per million years, representing an average of the most commonly used values (1.5% to 2.3%) for insect COI [33,45,46];

** microsatellite data [25] suggest only limited structure;

*** nuclear data [20] suggest population structure
